# Supplementary material for: Bcl-xL blockade targets neutrophils and synergizes with chemotherapy in lung squamous cell carcinoma
Source: EMBO Mol Med. 2026 Mar 31;18(5):1625–47. doi: 10.1038/s44321-026-00401-z (PMC13179379; doi:10.1038/s44321-026-00401-z)
Supplement: Supplementary file 1 — Appendix [file 44321_2026_401_MOESM1_ESM.pdf]

Appendix

Bcl-xL blockade targets neutrophils and synergizes with chemotherapy in lung squamous cell carcinoma

Table of Contents

Appendix Figure S1 | Gating strategy used for immune cell identification within tumors. 2

Appendix Figure S2 | Anti-PD-1 does not trigger major changes of the immune TME. 3

Appendix Figure S3 | Gating strategy used for identification of progenitor subsets within bone marrow and spleen. 4

Appendix Figure S4 | Bcl-xL blockade enhances the response of human squamous tumor cells to chemotherapy. 5

Appendix Figure S1

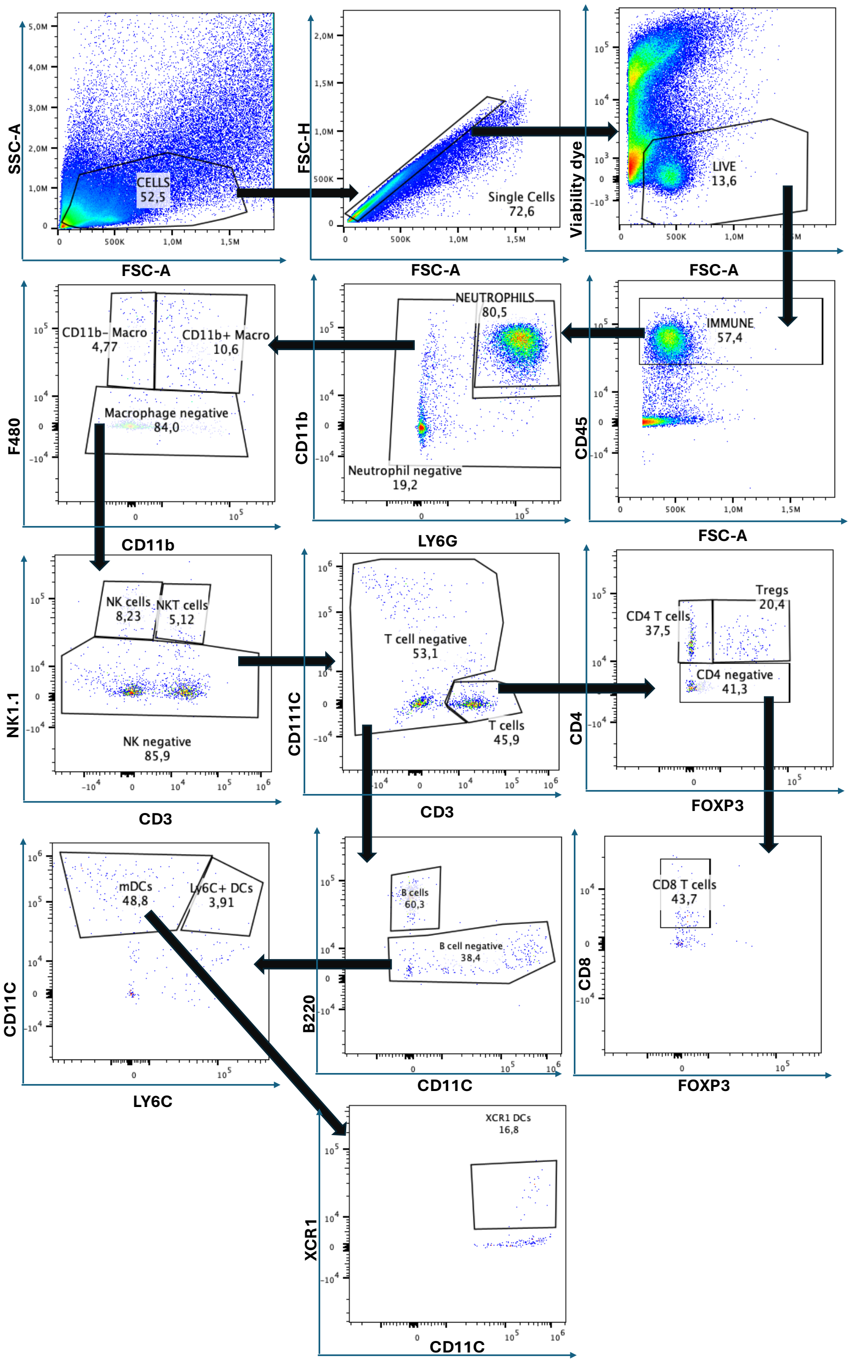

Appendix Figure S1 | Gating strategy used for immune cell identification within tumors. Debris was excluded with the FSC-A / SSC-A gate followed by inclusion of all single cells with FSC-A / FSC-H gate. Then, live cells were identified as being negative for viability dye. Finally, all immune cell types were identified by CD45 expression followed by identification of specific immune cell type based on their specific markers as shown in the gates - (**Neutrophil**: $LY6G^+$ / **B cells**: $B220^+$ / **$CD11b^+$ Macro**: $CD11b^+F480^+$ / **$CD11b^-$ Macro**: $CD11b^-F480^+$ / **NK cells**: $NK1.1^+$ / **NKT cells**: $NK1.1^+CD3^+$ / **CD4 T cells**: $CD3^+CD4^+$ / **CD8 T cells**: $CD3^+CD8^+$ **/ Tregs**: $CD3^+CD4^+FOXP3^+$ / **$XCR1^+$DCs**: $CD11c^+XCR1^+$ / **$LY6C^+$ DCs**: $CD11c^+LY6C^+$).

Appendix Figure S2

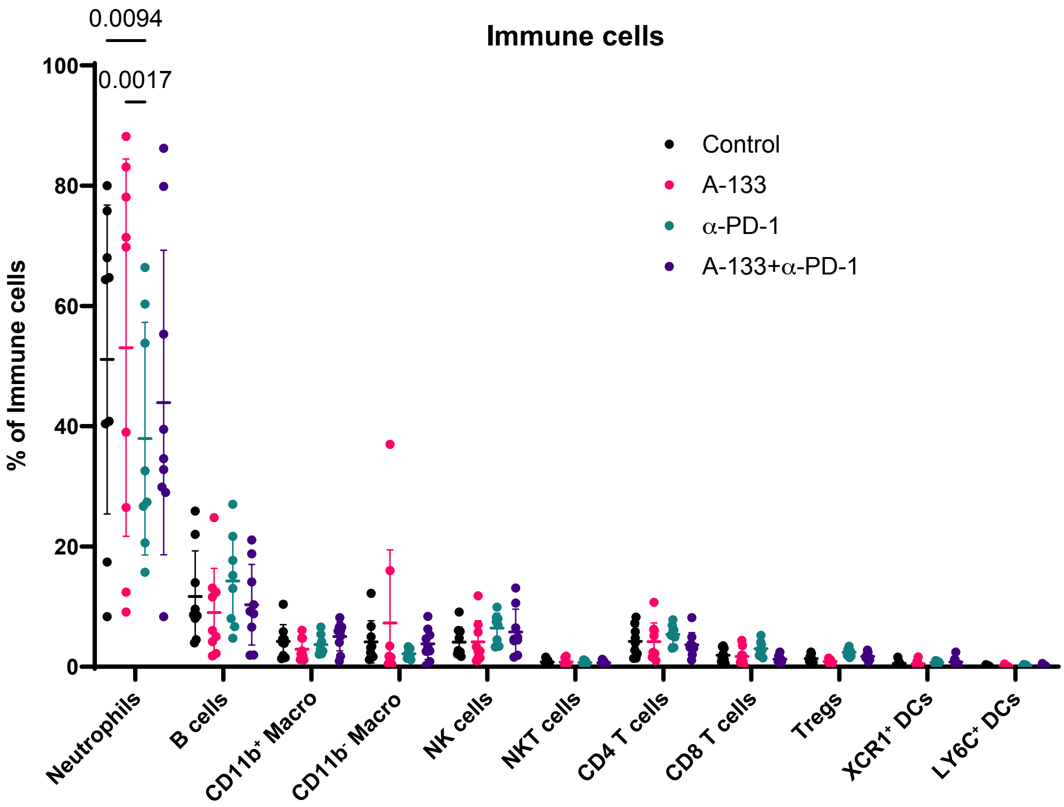

Appendix Figure S2 | Anti-PD-1 does not trigger major changes of the immune TME. Frequency of major immune cell populations among total CD45^{+} cells (n=8-9 tumors per group). p-value was determined using 2-way ANOVA. Data is shown as ±SD.

Appendix Figure S3

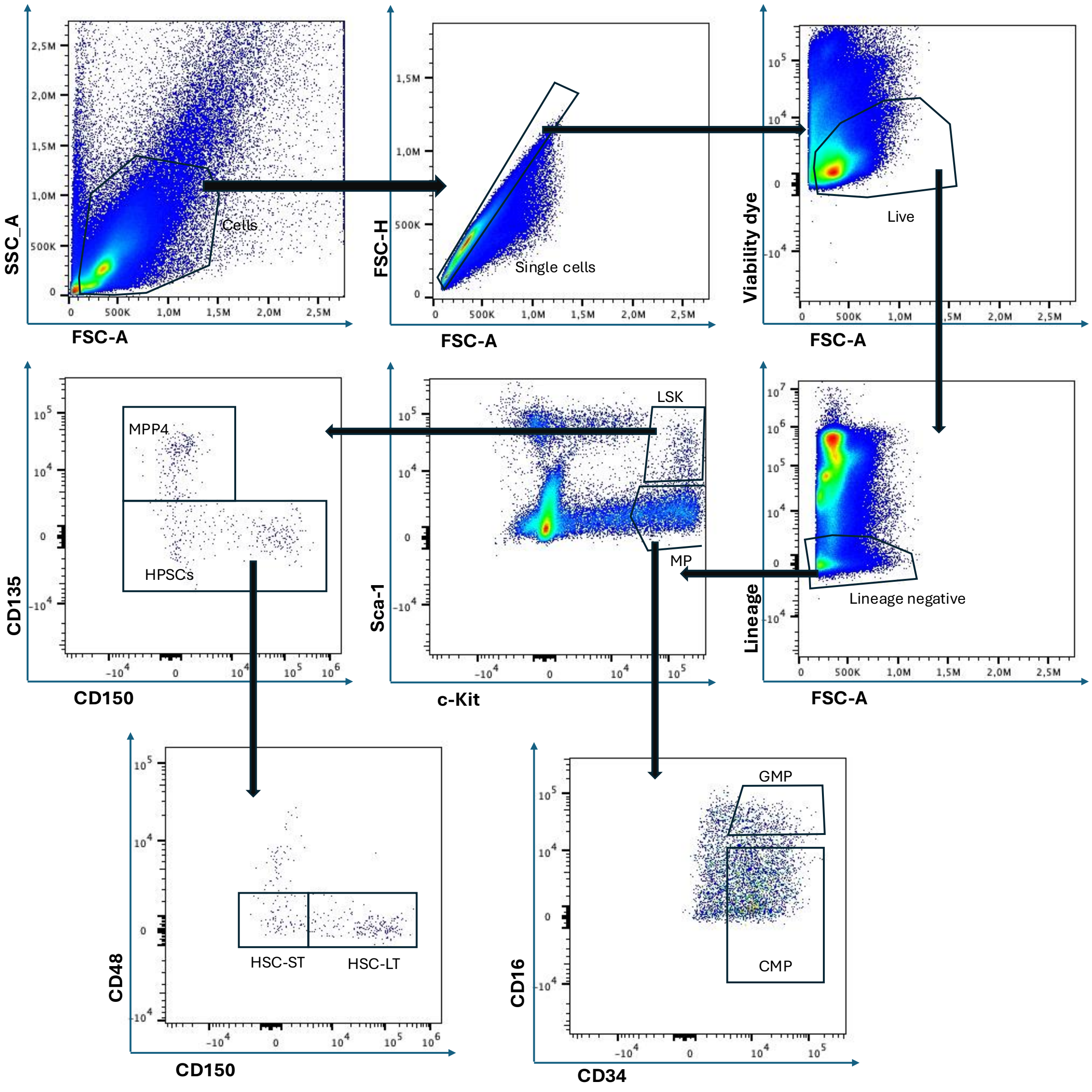

Appendix Figure S3 | Gating strategy used for identification of progenitor subsets within bone marrow and spleen. Debris was excluded with the FSC-A / SSC-A gate followed by inclusion of all single cells with FSC-A / FSC-H gate. Live cells were identified as being negative for viability dye. Then, all immune cells and RBCs were excluded based on their Lin^+ markers (TER119, B220, CD3, CD4, CD8, CD11b, and GR1). Lin^- cells were selected and were further gated for **LSK**: $c\text{-}kit^+Sca\text{-}1^+$ and **MP**: $c\text{-}kit^+Sca\text{-}1^-$. Within LSK^+ cells, identification of **MPP4**: $CD135^+CD150^-$ / **LT-HSC**: $CD135^-CD48^-CD150^+$ / **ST-HSC**: $CD135^-CD48^-CD150^-$. Within MP cells, identification of **GMP**: $CD16^+CD34^+$ / **CMP**: $CD16^-CD34^+$.

Appendix Figure S4

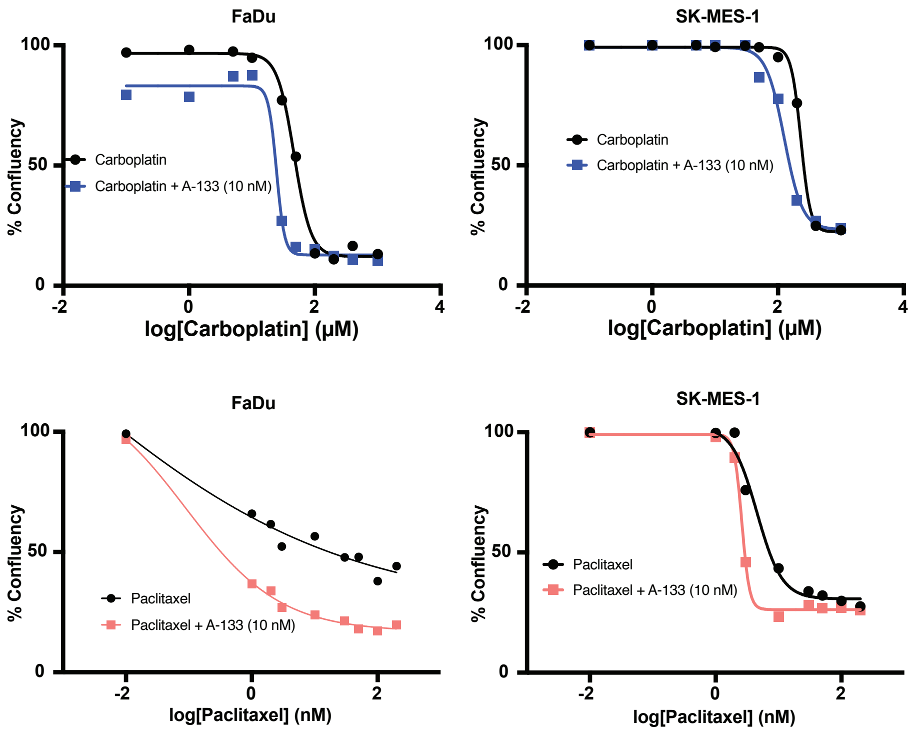

Appendix Figure S4 | Bcl-xL blockade enhances the response of human squamous tumor cells to chemotherapy. Logarithmic dose-response curve of cancer cell lines to chemotherapy alone or in combination with A-1331852. Data is shown as mean of two replicates fitted with a non-linear curve.
