# Supplementary material for: Bcl-xL blockade targets neutrophils and synergizes with chemotherapy in lung squamous cell carcinoma
Source: EMBO Mol Med. 2026 Mar 31;18(5):1625–47. doi: 10.1038/s44321-026-00401-z (PMC13179379; doi:10.1038/s44321-026-00401-z)
Supplement: Supplementary file 3 — Expanded View Figures [file 44321_2026_401_MOESM3_ESM.pdf]

Expanded View Figures

Figure EV1. Variability between TANs in LUSC compared to LUAD.

(**A**) (Left) Representative MPO staining and (Right) quantification in LUAD and LUSC lesions of SNL mice ($n = 11$ for LUAD and $n = 6$ for LUSC). Scale bars: 20 μm. (**B**) Comparison of frequency of $CD101^-$ (left), $CD14^+$ (middle) and PD-$L1^{hi}$ (right) TANs in KP ($n = 8$ tumors) and SNL ($n = 11$–14 tumors). (**C**) Heatmap depicting expression of individual genes by single-cell RNA-seq (scRNA-seq) of PBNs ($n = 1702$ cells) and TANs ($n = 445$ cells) from SNL mice. Genes are categorized by neutrophil subsets "N1", "N2", "N3", "N4", "N5", and "N6", as defined previously from murine scRNA-seq data (Data ref: Zilionis et al, 2019). (**D**) Split violin plots depicting expression of indicated neutrophil subset signature in scRNA-seq data of PBNs compared to TANs of SNL mice. Data information: *p*-value was determined using unpaired t-test for (**A**) and (**B** left and right), Welch's t-test for (**B** middle). For (**D**), Wilcoxon rank-sum test was used (****$p < 3.3E\text{-}0.5$). Data are shown as mean ± SD (**A**, **B**). For (**D**), box and whisker plot overlays depict median and upper and lower quartiles of expression. Each dot represents an individual cell.

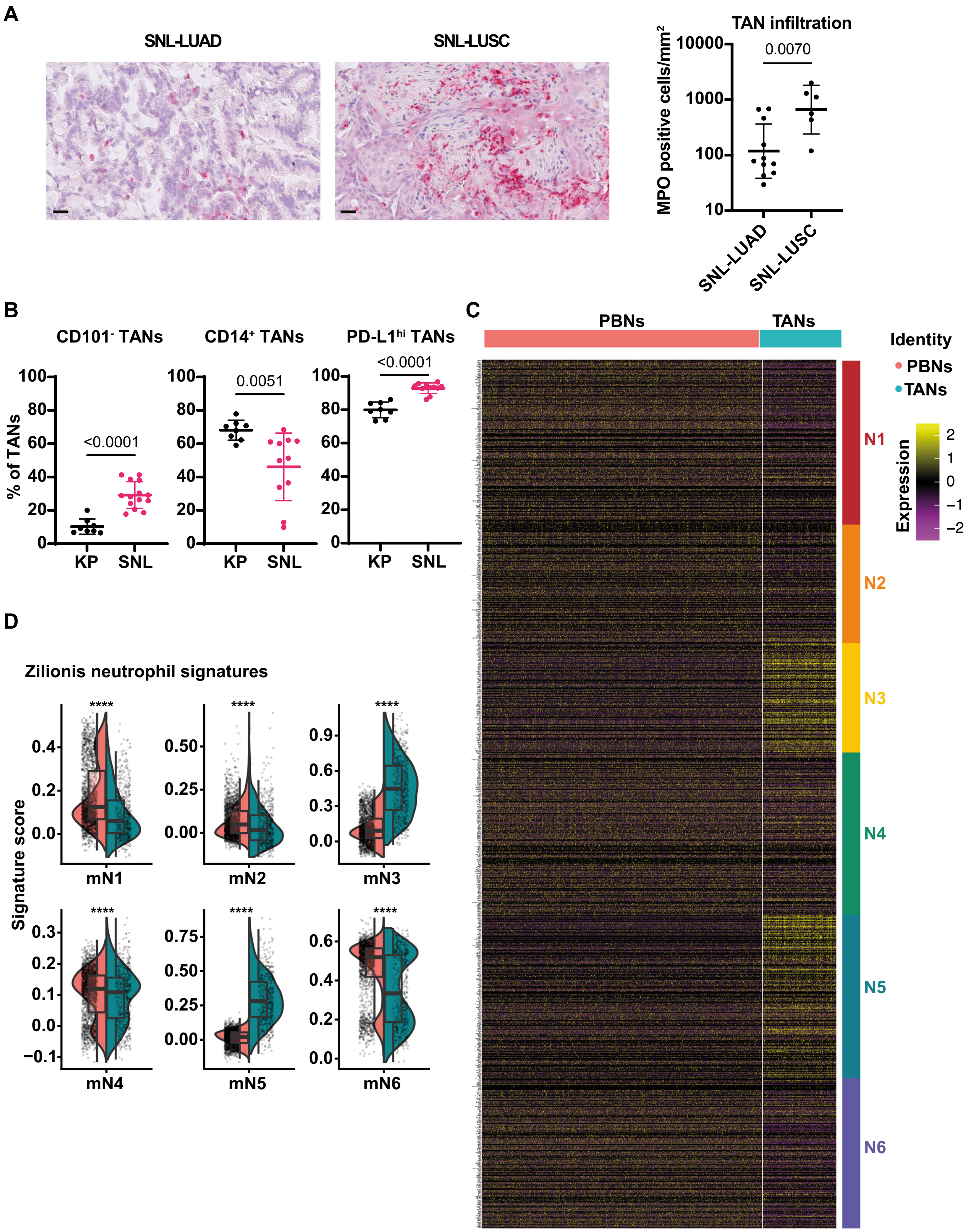
A
SNL-LUAD
SNL-LUSC
TAN infiltration
MPO positive cells/mm²
0.0070
SNL-LUAD
SNL-LUSC
B
CD101⁻ TANs
CD14⁺ TANs
PD-L1hi TANs
% of TANs
<0.0001
0.0051
<0.0001
KP
SNL
C
PBNs
TANs
Identity
PBNs
TANs
N1
N2
N3
N4
N5
N6
Expression
D
Zilionis neutrophil signatures
Signature score
mN1
mN2
mN3
mN4
mN5
mN6

 © The Author(s)

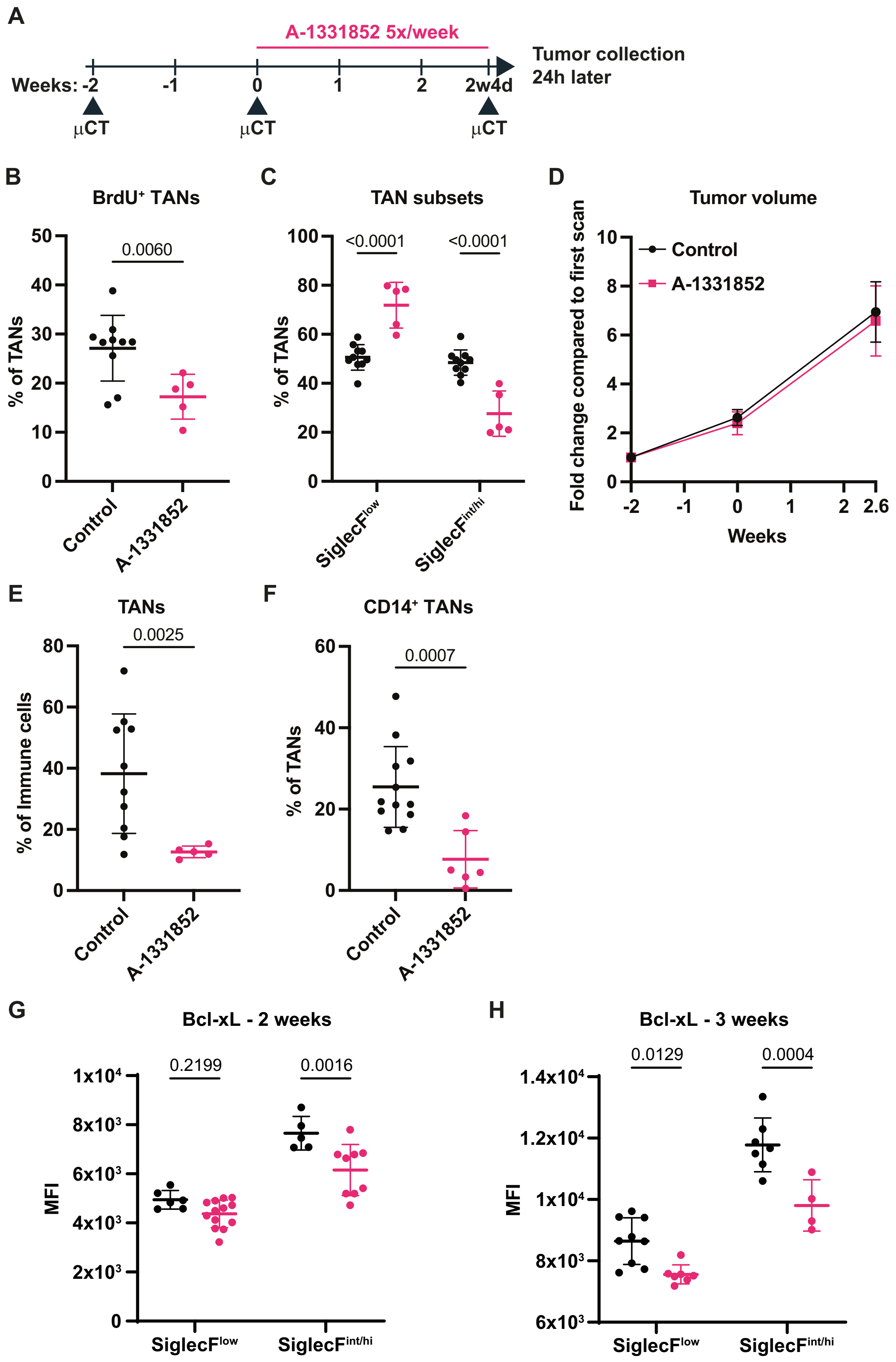
A
A-1331852 5x/week
Weeks: -2 -1 0 1 2 2w4d
Tumor collection 24h later
μCT μCT μCT
B
BrdU⁺ TANs
0.0060
% of TANs
Control
A-1331852
C
TAN subsets
<0.0001
<0.0001
% of TANs
SiglecF^{low}
SiglecF$^{int/hi}$
D
Tumor volume
Control
A-1331852
Fold change compared to first scan
Weeks
E
TANs
0.0025
% of Immune cells
Control
A-1331852
F
CD14⁺ TANs
0.0007
% of TANs
Control
A-1331852
G
Bcl-xL - 2 weeks
0.2199
0.0016
MFI
SiglecF^{low}
SiglecF$^{int/hi}$
H
Bcl-xL - 3 weeks
0.0129
0.0004
MFI
SiglecF^{low}
SiglecF$^{int/hi}$

Figure EV2. Bcl-xL blockade 5x/week does not diminish SNL tumor growth.

(**A**) Scheme of in vivo Bcl-xL blockade regimen. (**B**) Proportions of 6.5-day-old $BrdU^{+}$ TANs among total TANs ($n = 10$ for control and $n = 5$ for treated group). (**C**) Proportions of SiglecF TAN subsets among total TANs ($n = 10$ tumors for control and $n = 5$ for treated group). (**D**) Tumor growth kinetics measured by µCT ($n = 11$ tumors for control group and $n = 6$ tumors for treated group). (**E**) Proportions of TANs among all immune cells ($n = 10$ tumors for control and $n = 5$ for treated group). (**F**) Proportions of CD14 TANs among total TANs ($n = 12$ tumors for control and $n = 6$ tumors for treated). (**G**) MFI of Bcl-xL expression in $SiglecF^{low}$ ($n = 6$ tumors for control and $n = 13$ for treated group) and $SiglecF^{int/hi}$ ($n = 5$ for control and $n = 9$ for treated group) TANs. (**H**) MFI of Bcl-xL expression in $SiglecF^{low}$ ($n = 9$ tumors for control and $n = 7$ for treated group) and $SiglecF^{int/hi}$ ($n = 7$ for control and $n = 4$ for treated group) TANs. Data information: *p*-value was determined using Welch's t-test for (**B**), (**E**), and (**F**). For (**C**), (**D**), (**G**), and (**H**), 2-way ANOVA was used. Data are shown as mean ± SD except for (**D**), which is shown as mean ± SEM.

 © The Author(s)

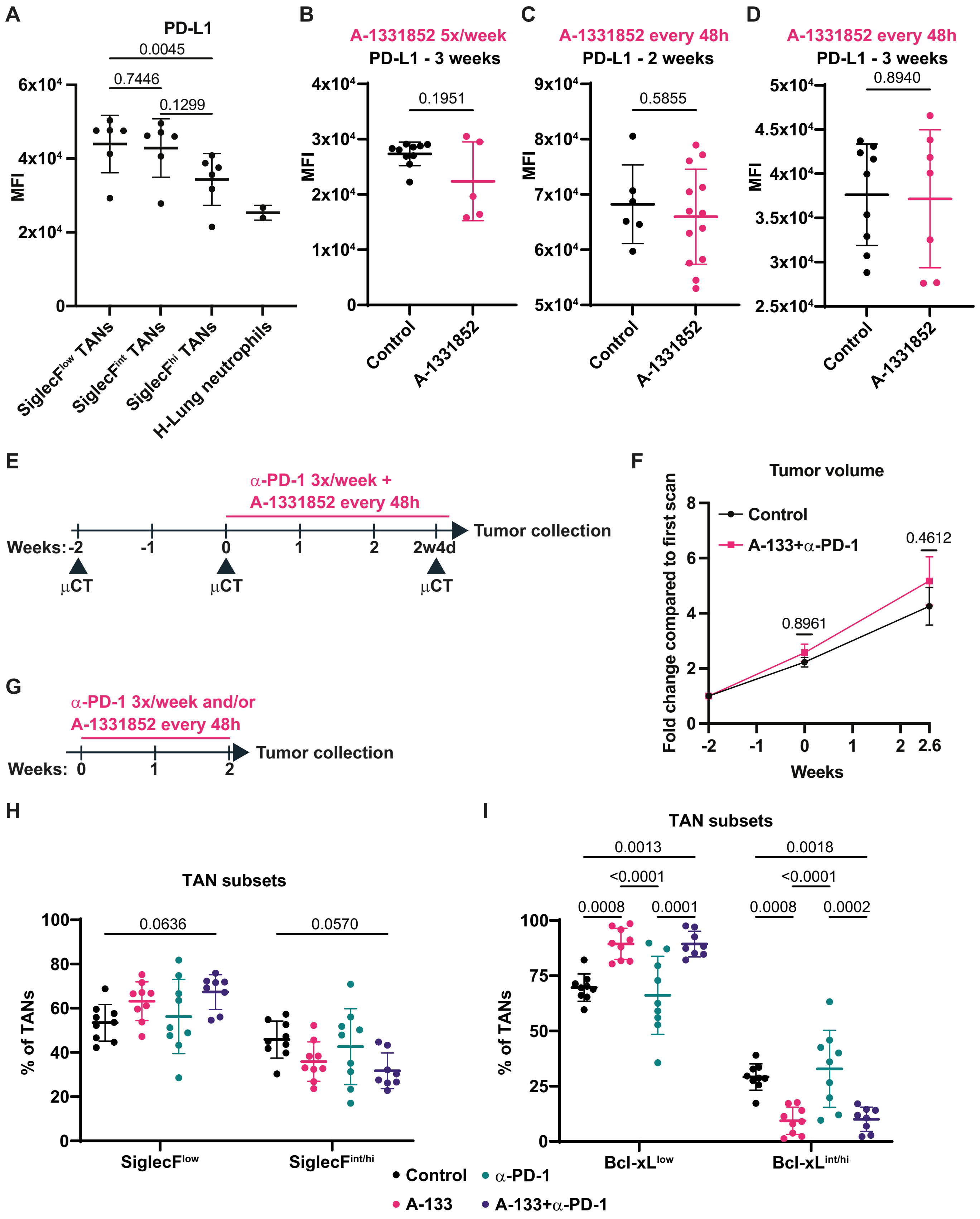

A
PD-L1
0.0045
0.7446
0.1299
MFI
SiglecF^low TANs
SiglecF^int TANs
SiglecF^hi TANs
H-Lung neutrophils
B
A-1331852 5x/week
PD-L1 - 3 weeks
0.1951
Control
A-1331852
C
A-1331852 every 48h
PD-L1 - 2 weeks
0.5855
D
A-1331852 every 48h
PD-L1 - 3 weeks
0.8940
E
α-PD-1 3x/week +
A-1331852 every 48h
Tumor collection
Weeks: -2 -1 0 1 2 2w4d
μCT
F
Tumor volume
Control
A-133+α-PD-1
0.4612
0.8961
Fold change compared to first scan
Weeks
G
α-PD-1 3x/week and/or
A-1331852 every 48h
Tumor collection
Weeks: 0 1 2
H
TAN subsets
0.0636
0.0570
% of TANs
SiglecF^low
SiglecF^int/hi
Control
α-PD-1
A-133
A-133+α-PD-1
I
TAN subsets
0.0013
<0.0001
0.0008
0.0001
0.0018
<0.0001
0.0008
0.0002
Bcl-xL^low
Bcl-xL^int/hi

◀ **Figure EV3. Combined Bcl-xL blockade and anti-PD-1 does not diminish tumor growth.**

(**A**) MFI of PD-L1 expression among TAN subsets ($n = 6$ for matched tumors and $n = 2$ for healthy lung (H-Lung) neutrophils). (**B**) MFI of PD-L1 expression in TANs upon 5x/week A-1331852 regimen for 3 weeks ($n = 10$ tumors for control and $n = 5$ for treated group). (**C**) MFI of PD-L1 expression in TANs upon 1x/48 h A-1331852 regimen for 2 weeks ($n = 6$ tumors for control and $n = 13$ for treated group). (**D**) MFI of PD-L1 expression in TANs upon 1x/48 h A-1331952 regimen for 3 weeks ($n = 9$ tumors for control and $n = 7$ for treated group). (**E**) Scheme of anti-PD-1 immunotherapy combined with A-1331852. (**F**) Tumor growth kinetics measured by µCT ($n = 12$ tumors for control group and $n = 8$ tumors for treated group). (**G**) Scheme of anti-PD-1, A-1331852 or their combination for 2 weeks. (**H**) Proportion of SiglecF TAN subsets among total TANs ($n = 8$–9 tumors per group). (**I**) Proportion of Bcl-xL TAN subsets among total TANs ($n = 8$–9 tumors per group). Data information: *p*-value was determined using Friedman test for (**A**). For (**B**), Welch t-test was used and for (**C**), and (**D**), unpaired t-test was used. For (**F**), (**H**), and (**I**), 2-way ANOVA was used. Data are shown as mean ± SD except for (**F**), which is shown as mean ± SEM.

 © The Author(s)

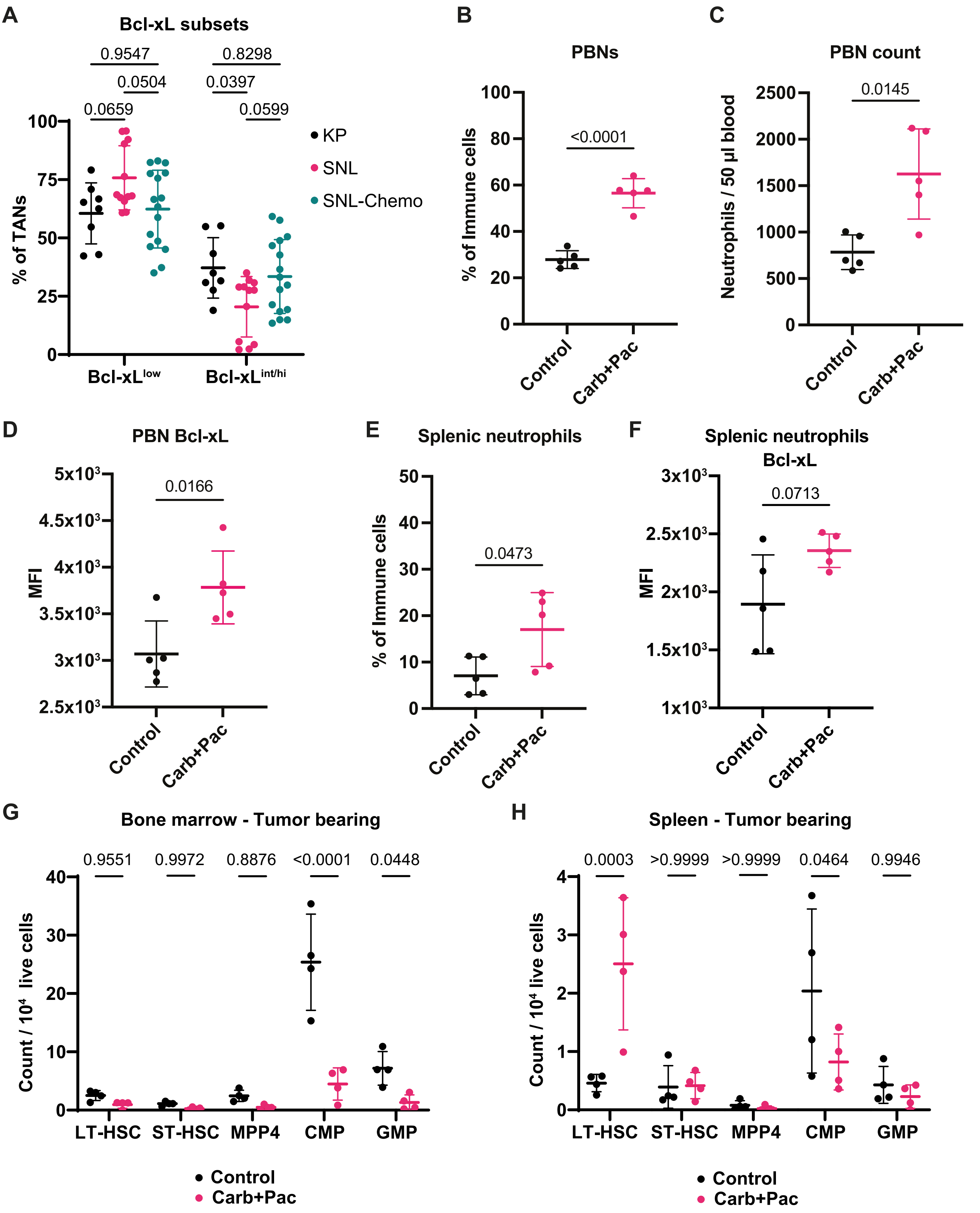
A
Bcl-xL subsets
0.9547
0.8298
0.0504
0.0397
0.0659
0.0599
% of TANs
KP
SNL
SNL-Chemo
Bcl-xL^low
Bcl-xL^int/hi
B
PBNs
<0.0001
% of Immune cells
Control
Carb+Pac
C
PBN count
0.0145
Neutrophils / 50 µl blood
Control
Carb+Pac
D
PBN Bcl-xL
0.0166
MFI
Control
Carb+Pac
E
Splenic neutrophils
0.0473
% of Immune cells
Control
Carb+Pac
F
Splenic neutrophils Bcl-xL
0.0713
MFI
Control
Carb+Pac
G
Bone marrow - Tumor bearing
0.9551
0.9972
0.8876
<0.0001
0.0448
Count / 10⁴ live cells
LT-HSC
ST-HSC
MPP4
CMP
GMP
Control
Carb+Pac
H
Spleen - Tumor bearing
0.0003
>0.9999
>0.9999
0.0464
0.9946
Count / 10⁴ live cells
LT-HSC
ST-HSC
MPP4
CMP
GMP
Control
Carb+Pac

© The Author(s)

Figure EV4. Combination chemotherapy increases circulating neutrophil numbers and their expression of Bcl-xL.

(**A**) Proportion of Bcl-xL TAN subsets among total TANs ($n = 8$–16 tumors per group). (**B**) Proportions of PBNs among immune cells in control group or carboplatin and paclitaxel (Carb+Pac) treated group. (**C**) Absolute PBN counts per 50 µl of blood. (**D**) MFI of Bcl-xL expression in PBNs. (**E**) Splenic neutrophil proportions among immune cells. (**F**) MFI of Bcl-xL expression in splenic neutrophils. (**G**, **H**) Quantities of hematopoietic precursors in the bone marrow (**G**) and spleen (**H**) among 10^4 live cells ($n = 4$ mice per group). Data information: *p*-value was determined using 2-way ANOVA for (**A**), (**G**), and (**H**). For (**B**), (**C**), (**D**), (**E**), and (**F**), Welch's t-test was used ($n = 5$ per group). Data are shown as mean ± SD.

© The Author(s)

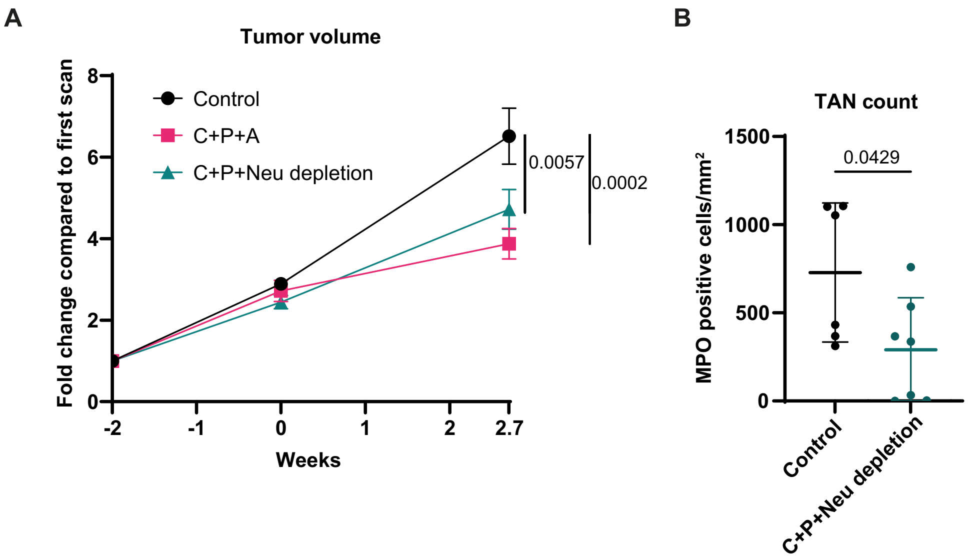

Figure EV5. Partial neutrophil depletion is comparable to A-1331852 in the anti-tumor response to chemotherapy.

(**A**) Tumor growth kinetics measured by μCT ($n = 9$–14 tumors per group). (**B**) IHC Quantification of MPO positive cells per mm^2 of tumor ($n = 6$ tumors for control and $n = 7$ for neutrophil depleted group). Data information: *p*-value was determined using 2-way ANOVA for (**A**) and unpaired t-test for (**B**). Data is shown as ±SEM for (**A**) and ±SD for (**B**).

© The Author(s)
